# The Relationships among the Urinary Iodine Concentration, Selenium Intake, and Thyroid Antibodies in Adults, Including the Interaction between Iodine and Selenium: National Health and Nutrition Examination Survey 2007–2012

**DOI:** 10.3390/nu16203443

**Published:** 2024-10-11

**Authors:** Chenyu Zhang, Haoyu Wang, Weiping Teng, Zhongyan Shan

**Affiliations:** Department of Endocrinology and Metabolism, Institute of Endocrinology, National Health Commission (NHC) Key Laboratory of Diagnosis and Treatment of Thyroid Diseases, The First Affiliated Hospital of China Medical University, China Medical University, Shenyang 110001, China; zhangchenyu@cmu.edu.cn (C.Z.); hywang.endocr@cmu.edu.cn (H.W.); twp@vip.163.com (W.T.)

**Keywords:** iodine, selenium, TPOAb, TgAb, thyroid autoimmunity

## Abstract

Objectives: The objective of this study was to examine the urinary iodine concentration (UIC)–thyroid autoimmunity (TAI) association and UIC–selenium intake interaction in U.S. adults. Methods: We analyzed 2007–2012 National Health and Nutrition Examination Survey (NHANES) data on ≥20-year-old adults (*n* = 6612). Their food and supplemental selenium intake was measured. The associations of the UIC and selenium intake with thyroid peroxidase antibody (TPOAb) positivity, thyroglobulin antibody (TgAb) positivity, and TAI were assessed via weighted multivariable logistic regression. Interaction and subgroup analyses were conducted. Nonlinear relationships were explored and visualized via restricted cubic splines (RCSs). Results: Compared with a UIC 100~200 μg/L, a UIC 500~800 μg/L was associated with a 57% increased TPOAb positivity risk (OR = 1.57 [CI = 1.07–2.30]; *p* = 0.022), a one-fold greater TgAb positivity risk (OR = 2.00 [CI = 1.10–3.65]; *p* = 0.025), and a 62% increased TAI risk (OR = 1.62 [CI = 1.07–2.45]; *p* = 0.024). Nonlinear relationships between the UIC and thyroid antibody positivity were observed. According to the univariate models, each 1 μg increase in selenium intake was associated with a 0.049 IU/mL decrease in the TPOAb levels (β [95% CI] = −0.049 [−0.092–−0.005]; *p* = 0.028). In the low-selenium group, a UIC of 200~300 μg/L was a risk factor for TPOAb positivity (*p* = 0.046). At a moderate level of selenium intake, a UIC of 300~800 μg/L significantly increased the TPOAb positivity risk (all *p* < 0.05). At a high level of selenium intake, the UIC and TPOAb positivity risks were not significantly associated (all *p* > 0.05). Conclusions: A UIC of 500~800 μg/L is an independent TAI risk factor. The selenium intake modifies the UIC–thyroid antibody positivity relationship, with the association disappearing at high selenium levels.

## 1. Introduction

Iodine is a vital trace element for thyroid health, with studies showing a U-shaped relationship between the urinary iodine concentration (UIC) and thyroid disorders [1,2]. The thyroid peroxidase antibody (TPOAb) and thyroglobulin antibody (TgAb) are key antibodies in autoimmune thyroid disease (AITD). A large cross-sectional study in China revealed that iodine deficiency is a risk factor for the development of thyroid autoimmunity (TAI) [3]. A five-year longitudinal study indicated that both exceedingly low and exceedingly high iodine intake levels are associated with an increased prevalence of TAI [4]. To optimize iodine nutrition and protect thyroid health, many iodine-deficient countries have implemented voluntary or mandatory salt iodization programs. However, concerns have been raised about the impact of iodine intake on the risk of developing TAI. AITD represents the most common cause of hypothyroidism in regions where iodine is in sufficient supply [5]. Thus, the relationship between iodine intake and thyroid antibody positivity and the safety range of iodine supplementation in relation to TAI have become key research focuses.

Selenium is also an essential trace element that is crucial for maintaining thyroid health. Selenoproteins, such as deiodinases involved in thyroid hormone metabolism and glutathione peroxidases (GPx) related to thyroid oxidative stress, play key roles in thyroid function [6]. Some studies have highlighted potential links between iodine and selenium supplementation in thyroid physiology. In patients with severe iodine deficiency (cretinism), selenium supplementation for six months resulted in significant hypothyroidism [7]. This may have been due to selenium’s activation of extrathyroidal deiodinases, which led to further iodine loss through the kidneys and feces, worsening the iodine deficiency [7]. In animal studies, selenium-deficient rats fed with high iodine developed TNF-β-mediated thyroid fibrosis [8], which was significantly reduced with selenium supplementation [8]. Excessive iodine induces NOS and ROS damage in thyroid tissue, but this damage is mitigated when selenium levels are adequate, likely due to the protective role of increased selenoprotein expression [9]. Limited clinical and experimental studies suggest that there needs to be a balance between iodine and selenium supplementation.

Similar to iodine, selenium is essential for TAI. In pathological thyroid sections from patients with Hashimoto’s thyroiditis (HT), Gpx3 remains highly expressed in residual follicles, but its expression is absent in infiltrating lymphocytes and fibrotic structures [10]. Insufficient selenium storage promotes oxidative stress, which triggers autoimmunity [6,11], while selenium supplementation helps prevent or delay the onset of HT and reduces the TPOAb levels [12,13]. This raises the question of how selenium intake might alter the safe range of iodine intake for preventing TAI. Large-scale, high-quality, cross-sectional studies have not investigated the relationship between iodine and thyroid antibodies across different selenium intake subgroups. Existing studies have only adjusted for iodine or selenium as confounders, without exploring their interaction. In the present study, we used National Health and Nutrition Examination Survey (NHANES) 2007–2012 data to examine the link between the UIC and thyroid antibody positivity in U.S. adults and to determine whether selenium intake affects this relationship.

## 2. Materials and Methods

### 2.1. Design

The NHANES is a cross-sectional survey that employs complex, stratified, multistage probability sampling to provide a representative sample of the noninstitutionalized U.S. population, assessing their health and nutritional status [14]. This study analyzed data from three NHANES cycles (2007–2012) that included thyroid function tests, the UIC, and the selenium intake (at least 24 h of dietary selenium intake) [14]. Demographic, examination, and questionnaire data were also included. The NHANES protocols were approved by the Ethics Review Board of the National Center for Health Statistics, and all the participants provided written informed consent. The data are available on the NHANES website (http://www.cdc.gov/nchs/nhanes.htm, accessed on 10 March 2024 [14].

### 2.2. Study Population

**Figure 1:**
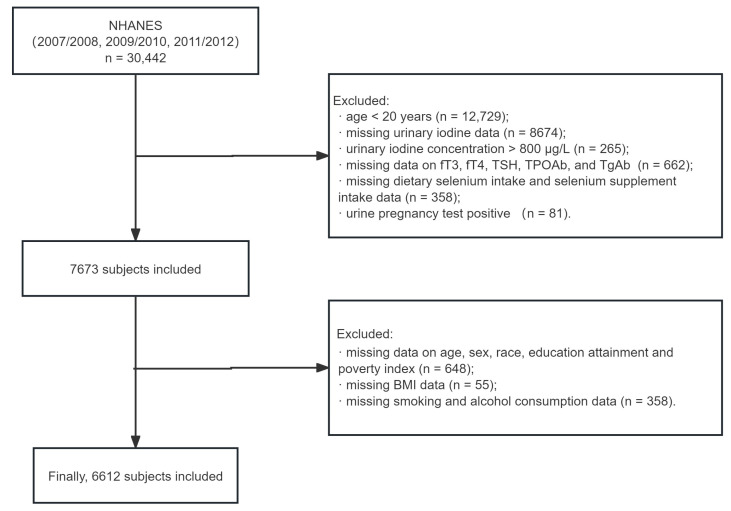
Flowchart of the study population.

In the present study, we included all nonpregnant participants aged ≥20 years with a UIC ≤ 800 µg/L, excluding those with a positive pregnancy test, who had complete thyroid function data (including free thyroxine [fT4], free triiodothyronine [fT3], thyroid-stimulating hormone [TSH], and TPOAb and TgAb levels). Their selenium intake from diet and supplements, along with key confounders (age, sex, race/ethnicity, the poverty index, education level, body mass index [BMI], smoking status, and alcohol consumption status), were also collected. A total of 6612 participants with complete data were included in the analysis, as illustrated in Figure 1. Of the 15,983 participants of the NHANES aged 20 years and older with complete data for the weight variable “WTDRD1”, it was assumed that these participants represented the overall U.S. population aged 20 years and older. The 6612 participants included in our study did not differ significantly in age, sex, education level, smoking status, alcohol consumption status, BMI, TSH levels, TPOAb positivity, TgAb positivity, or UIC from the overall 15,983 participants or the 9371 who were not included (all *p* > 0.05; Supplementary Table S1). However, the included participants were more likely to be non-Hispanic White, to be from higher-income households, and to have higher fT4 and fT3 levels (all *p* < 0.05; Supplementary Table S1). Owing to the exclusion of participants with a UIC > 800 µg/L, the mean UIC was lower in the study group (*p* < 0.05). Therefore, while the results may not be generalizable to the entire U.S. population aged ≥20 years, they still retain some representativeness.

### 2.3. Study Variables

- Thyroid Function and Antibodies

Blood samples for the thyroid function tests were processed, stored, and shipped to the University of Washington, Seattle, WA, USA (2007/2008, 2009/2010), and to the Collaborative Laboratory Services Center in Ottumwa, IA, USA (2011/2012). The TPOAb and TgAb titers were measured with a two-step sequential immunoenzymatic “sandwich” assay. The TSH levels were measured via a third-generation two-site immunoassay sandwich. The fT3 levels were measured with a competitive binding immunoassay. The fT4 levels were measured with a two-step immunoassay. All the assays were performed with the Access 2 immunoassay system (Beckman Coulter). The normal reference range provided by the assay kit was <9.0 IU/mL for TPOAb and <4.0 IU/mL for TgAb.

- The UIC

The urine samples were processed, stored, and shipped to the Division of Laboratory Sciences, National Center for Environmental Health. The UIC was measured via inductively coupled plasma–dynamical reaction cell mass spectrometry (ICP-DRC-MS). The UIC was categorized into five groups: <100 µg/L, 100~200 µg/L, 200~300 µg/L, 300~500 µg/L, and 500~800 µg/L [15,16]. According to the WHO, a UIC of 100–200 µg/L is considered to indicate adequate iodine intake [15].

- Selenium Intake

During each NHANES cycle, the participants provided two 24 h food recalls that were used to estimate their energy, nutrient, and other dietary intakes. The first dietary recall was collected during the NHANES visit, and the second was collected by telephone 3 to 10 days later. We averaged the estimated dietary selenium intake from the two recalls (or used the first recall if only one was available). The dietary supplement use was also averaged or based on one day if needed. The total selenium intake was calculated as the summed (or averaged) dietary selenium intake and supplemental selenium intake. For the analysis, the selenium intake was categorized into tertiles based on population percentiles: T1 (<88.95 µg), T2 (88.95~133.60 µg), and T3 (≥133.60 µg).

- Other Variables

The covariates in this study included continuous variables (age (years) and selenium intake (µg)) and categorical variables (sex (men/women), race/ethnicity (non-Hispanic White, non-Hispanic Black, Mexican American, other Hispanic, and other/multiracial), BMI (underweight: <18.5 kg/m^2^; normal: 18.5 to <25 kg/m^2^; overweight: 25 to <30 kg/m^2^; obese: 30 kg/m^2^ or greater), family income-to-poverty ratio (≤1.3; 1.3~3.5; ≥3.5), education level (less than 9th grade; 9–11th grade; high school grad/GED; some college or AA degree; college graduate or higher), self-reported alcohol consumption status (non-drinker; 1–5 drinks/month; 5–10 drinks/month; and 10+ drinks/month), and smoking status (non-smoker: smoked fewer than 100 cigarettes in lifetime; former smoker: smoked at least 100 cigarettes, but does not currently smoke; current smoker: smoked at least 100 cigarettes and currently smokes daily)).

### 2.4. Data Analysis

After stratifying the UIC, we used weighted mean *t*-tests or median Mann–Whitney tests, and chi-squared tests for categorical data, to assess and compare the demographic and covariate information between the groups. The continuous and categorical variables are presented as the mean ± SE or median (IQR) and percentage, respectively. Weighted logistic regression models were used to estimate the odds ratios (ORs) and 95% confidence intervals (95% CIs) for the associations of the UIC with the TPOAb positivity, TgAb positivity, and TAI. Confounding factors were adjusted for in three models: Model 1 was unadjusted; Model 2 was adjusted for age and sex; and Model 3 was a fully adjusted model, controlling for age, sex, race/ethnicity, poverty index, education level, BMI, selenium intake, smoking status, and alcohol consumption status. Next, we divided the participants into tertiles based on their selenium intake. Weighted linear regression was used to investigate the relationship between the selenium intake and the TPOAb and TgAb levels, and weighted logistic regression was used to examine the relationship between different levels of selenium intake and the risk of developing thyroid antibody positivity. Subgroup analyses and interaction effects between the selenium intake and the UIC on thyroid antibody positivity were explored using weighted linear and logistic regression. A restricted cubic spline (RCS) analysis was employed to examine nonlinear relationships. All the analyses were conducted using R (version 4.3.2) and the “survey” package, accounting for NHANES’s complex survey design. The statistical significance was set at 0.05, except for the interaction analyses, where *p* < 0.10 was considered to indicate statistical significance.

## 3. Results

This study included 6612 participants. Their demographic characteristics are presented in Table 1. After UIC stratification, significant differences were observed across UIC groups in sex (*p* < 0.001), household income (*p* = 0.009), smoking status (*p* = 0.002), BMI (*p* = 0.001), selenium intake (*p* < 0.001), and TgAb positivity (*p* = 0.029).

### 3.1. Associations between the UIC and Thyroid Antibody Positivity

**Figure 2:**
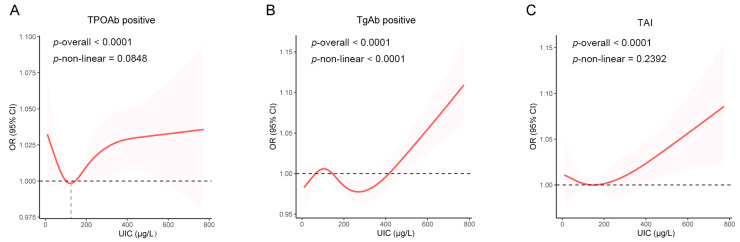
Dose–response relationship using a restricted cubic spline in the overall population. Age, sex, race/ethnicity, poverty index, education level, BMI, selenium intake, smoking status, and alcohol consumption status were adjusted for. (**A**) Association between the UIC and the risk of developing TPOAb positivity; (**B**) Association between the UIC and the risk of developing TgAb positivity; (**C**) Association between the UIC and the risk of developing TAI.

Weighted logistic regression was used to investigate the relationships between the UIC and the risk of developing TPOAb positivity, TgAb positivity, or TAI. A UIC of 500~800 µg/L was associated with a significantly increased risk of developing thyroid antibody positivity (Table 2). After adjusting for confounders, individuals with a UIC of 500~800 µg/L had a 1.57-fold greater likelihood of developing TPOAb positivity (Model 3: OR = 1.57 [CI = 1.07~2.30]; *p* = 0.022), a 2.00-fold greater likelihood of developing TgAb positivity (Model 3: OR = 2.00 [CI = 1.10~3.65]; *p* = 0.025), and a 62% greater risk of developing TAI (Model 3: OR = 1.62 [CI = 1.07~2.45]; *p* = 0.024) than did those with a UIC of 100~200 µg/L (Table 2). However, a UIC < 100 µg/L or 200~500 µg/L did not significantly increase the risk of developing thyroid antibody positivity (all *p* > 0.05; Table 2). The dose–response relationship between the UIC and the risk of developing thyroid antibody positivity via the RCS analysis (adjusted for Model 3 covariates) is presented in Figure 2. The shaded area represents the 95% confidence interval, indicating the range of changes in the TPOAb positivity risk at different UIC levels. A nonsignificant trend toward a U-shaped relationship between the UIC and the risk of developing TPOAb positivity was observed (*p* overall < 0.001; *p* nonlinear = 0.085; Figure 2A), with the lowest TPOAb positivity risk observed for participants with a UIC of 121.02 µg/L. At lower UIC levels, the risk of TPOAb positivity decreased as the UIC increased; however, when the UIC exceeded 121.02 μg/L, the risk of TPOAb positivity gradually increased or leveled off. A significant J-shaped relationship was observed between the UIC and the risk of developing TgAb positivity (*p* overall < 0.001; *p* nonlinear = 0.043), with the risk of TgAb positivity increasing as UIC rose above 261.59 μg/L (Figure 2B). There was no significant nonlinear relationship between the UIC and TAI risk (*p* overall < 0.001; *p* nonlinear = 0.272; Figure 2C).

### 3.2. Selenium Intake Stratification

The selenium intake was divided into three tertiles: low (T1), moderate (T2), and high (T3). The characteristics of the participants in each tertile are described in Table 3. Significant between-tertile differences were observed in age (*p* = 0.030), sex (*p* < 0.001), race/ethnicity (*p* = 0.004), education level (*p* < 0.001), household income (*p* < 0.001), smoking status (*p* < 0.001), alcohol consumption status (*p* < 0.001), fT3 level (*p* = 0.006), TgAb positivity (*p* = 0.011), and UIC (*p* < 0.001).

### 3.3. Associations between Selenium Intake and Thyroid Antibody Levels

**Figure 3:**
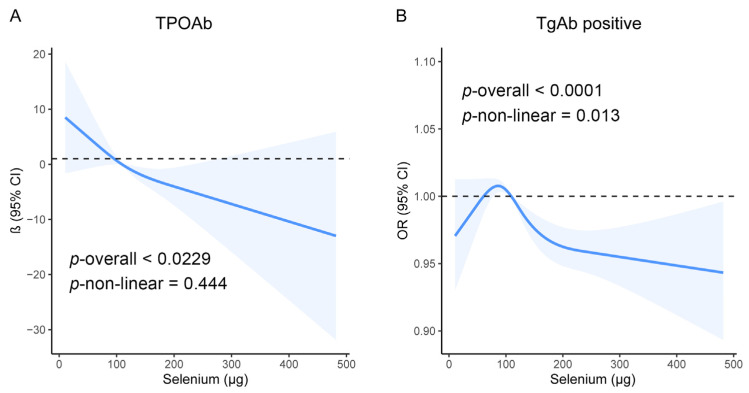
Dose–response relationship using a restricted cubic spline in the overall population. An unadjusted model was used. (**A**) Association between selenium intake and TPOAb levels; (**B**) Association between selenium intake and TgAb levels.

The weighted linear regression analysis revealed a significant inverse relationship between the selenium intake and the TPOAb levels. For every 1 µg increase in the selenium intake, the TPOAb levels decreased by 0.049 IU/mL (Model 1: β [95% CI] = −0.049 [−0.092–−0.005]; *p* = 0.028; Table 4). However, this relationship was no longer significant after adjusting for confounders (Table 4). The RCS analysis revealed no nonlinear relationship between the selenium intake and the TPOAb levels (*p* overall = 0.023; *p* nonlinear = 0.444; Figure 3A). There was no significant linear relationship between the selenium intake and the TgAb levels, regardless of the adjustment (all *p* > 0.05; Table 4). However, the RCS analysis revealed a significant inverted U-shaped relationship between the selenium intake and the risk of developing TgAb positivity (*p* overall < 0.001; *p* nonlinear = 0.013; Figure 3B). Logistic regression after selenium stratification revealed that a high selenium intake (T3) slightly reduced the risk of developing TgAb positivity in the crude model (Model 1: OR [95% CI] = 0.966 [0.941, 0.992]; *p* = 0.012; Table 4), but this association disappeared after adjusting for confounders (all *p* > 0.05; Table 4).

### 3.4. Interaction and Subgroup Analysis

**Figure 4:**
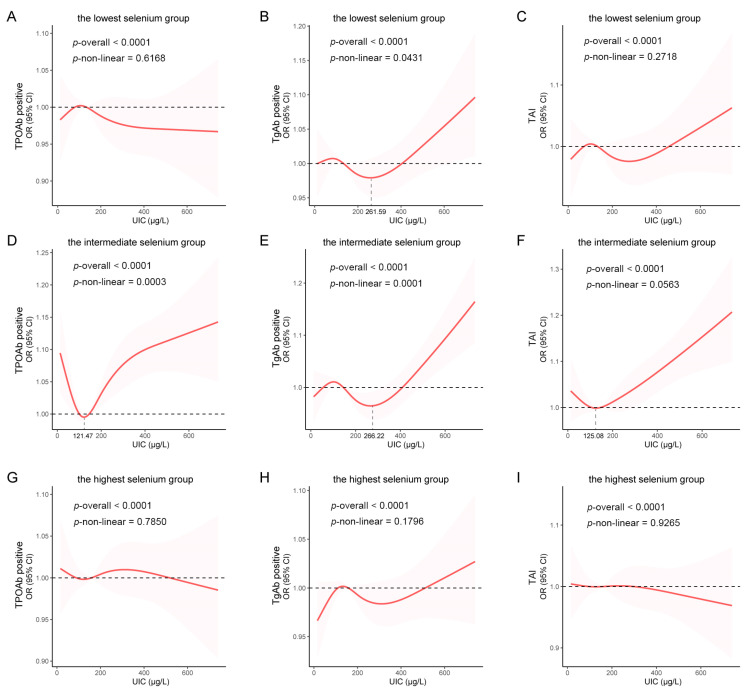
Dose–response relationship using a restricted cubic spline in populations stratified by different selenium intake levels. (**A**) Association between the UIC and the risk of developing TPOAb positivity in the lowest selenium group; (**B**) Association between the UIC and the risk of developing TgAb positivity in the lowest selenium group; (**C**) Association between the UIC and the risk of developing TAI in the lowest selenium group; (**D**) Association between the UIC and the risk of developing TPOAb positivity in the intermediate selenium group; (**E**) Association between the UIC and the risk of developing TgAb positivity in the intermediate selenium group; (**F**) Association between the UIC and the risk of developing TAI in the intermediate selenium group; (**G**) Association between the UIC and the risk of developing TPOAb positivity in the highest selenium group; (**H**) Association be-tween the UIC and the risk of developing TgAb positivity in the highest selenium group; (**I**) Association between the UIC and the risk of developing TAI in the highest selenium group.

The interaction analysis revealed a significant interaction effect between the selenium intake and the UIC on the risk of developing TPOAb positivity (all *p*-values for interaction < 0.10), but not on the risk of developing TgAb positivity or TAI (all *p*-values for interaction > 0.10; Table 5). The subgroup analysis revealed that, in the low-selenium group, a UIC of 200~300 µg/L was a risk factor for the development of TPOAb positivity (Table 5). In Model 3, those with a UIC of 200~300 µg/L had a 1.91-fold greater likelihood of developing TPOAb positivity than those with an adequate UIC (OR [95% CI] = 1.91 [1.01–3.61]; *p* = 0.046; Table 5). In the moderate-selenium group, a UIC of 300~800 µg/L was a risk factor for the development of TPOAb positivity (Table 5). After adjusting for confounders, a UIC of 300~500 µg/L increased the risk of developing TPOAb positivity by 82% (Model 3: OR = 1.82 [1.11–2.97]; *p* = 0.019), while a UIC of 500–800 µg/L was associated with a 2.6-fold greater risk (Model 3: OR = 2.60 [1.46~4.63]; *p* = 0.002; Table 5). The RCS analysis of the moderate-selenium group revealed the most pronounced U-shaped relationship between the UIC and the risk of developing TPOAb positivity (*p* overall < 0.001; *p* nonlinear < 0.001; Figure 4D). In the high-selenium group, no significant association was found between the UIC and the risk of developing TPOAb positivity (Table 5), and no nonlinear relationship was observed in the RCS analysis (*p* overall < 0.001; *p* nonlinear = 0.785; Figure 4G). In both the low- and moderate-selenium groups, the UIC showed a significant J-shaped relationship with the TgAb positivity (low-selenium group: *p* overall < 0.001; *p* nonlinear = 0.043; moderate-selenium group: *p* overall < 0.001; *p* nonlinear < 0.001; Figure 4B,E).

Other variables, including sex, an age > 45 years, race, BMI, the education level, the smoking status, the alcohol consumption status, and the poverty index, did not significantly interact with the UIC for thyroid antibody positivity (all *p*-values for interactions > 0.10; Supplementary Table S2). These factors independently affected the TPOAb and TgAb positivity and TAI risk without relying on changes in the UIC.

## 4. Discussion

Both iodine and selenium can influence thyroid antibody positivity, but large-scale studies on how selenium intake affects this relationship are limited. In this cross-sectional analysis, we found that a UIC of 500~800 µg/L significantly increased the risk of developing TPOAb positivity, TgAb positivity, and TAI. While the selenium intake alone did not affect the thyroid antibody levels after adjusting for confounders, we observed an interaction between the selenium intake and the UIC. Specifically, at a low level of selenium intake, a UIC of 200~300 µg/L increased the risk of developing TPOAb positivity; at a moderate level of selenium intake, a UIC of 300–800 µg/L significantly increased this risk; and at a high level of selenium intake, an association between the UIC and thyroid antibody positivity was not observed. Additionally, in the moderate-selenium group, we identified a U-shaped relationship between the UIC and the risk of developing TPOAb positivity and a J-shaped relationship with TgAb positivity. This study is the first to compare the relationships between the UIC and thyroid antibody positivity across different selenium intake levels.

The relationship between iodine intake and thyroid antibody positivity has been widely studied, yet the findings are mixed. In NOD.H-24 mice, excessive iodine intake leads to thyroid follicular epithelial damage and triggers an antigen–antibody response, a key mechanism in TAI [17]. In human studies, iodine supplementation appears to increase the exposure of Tg-hidden antigens [18]. In iodine-deficient regions, the thyroid antibody positivity rates significantly increase 5 to 15 years after iodine supplementation [19,20,21]. However, other studies suggest that an increased iodine intake has no impact on the risk of developing TAI [19,22,23]. In China (iodine-sufficient areas), the prevalence of TPOAb positivity actually decreases with increasing UIC [3]. These differences highlight that the effect of iodine supplementation on thyroid antibody positivity varies depending on an individual’s iodine status (e.g., deficiency or sufficiency). This can be explained by the nonlinear relationship between thyroid antibody levels and the UIC. Importantly, our findings suggest that these differences may be closely related to the selenium status of different populations. Selenium, an essential component of enzymes such as GPx and iodothyronine deiodinases [24,25], plays a crucial role in removing hydrogen peroxide (H2O2), thus influencing the TPO-mediated iodination of thyroglobulin [25,26]. Iodine-deficiency-induced thyroid hyperplasia can decrease GPx activity, leading to thyroid damage [27], whereas selenium supplementation can significantly increase GPx activity, thereby protecting the thyroid. In addition to its enzymatic antioxidant functions, selenium regulates autoimmunity by inhibiting pro-inflammatory cytokines through the Nrf2/ARE pathway [28]. In thyroid tissue slices from selenium-deficient HT patients, a marked reduction in GPx3 expression in lymphocytes infiltrating the thyroid tissue has been observed [10], indicating selenium’s protective role against TAI. Moreover, endoplasmic reticulum (ER) selenoproteins such as DIO2 participate in regulating ER stress and inhibiting apoptosis [29]; in cell and animal studies, an excessive iodine intake has been shown to activate ER stress-related proteins in thyroid follicular epithelial cells, such as IRE1α, XBP1, and PERK [30,31]. Selenium’s regulation of ER stress is another potential molecular mechanism through which it protects the thyroid from iodine-induced thyroid disease. The close physiological connection between iodine and selenium is key to explaining their interaction.

In our study, the RCS curves indicated that UICs of 121.02 µg/L and 261.59 µg/L were associated with the lowest risks of TPOAb and TgAb positivity, respectively. Weighted multivariable logistic regression revealed a significant increase in the thyroid antibody positivity risk only when the UIC exceeded 500 µg/L. Currently, a UIC of 100~200 µg/L is considered adequate for iodine nutrition and is included in the optimal UIC range associated with minimal TPOAb positivity risk. Our findings suggest that maintaining the UIC below 300 µg/L could minimize the risk of developing thyroid antibody positivity, particularly TgAb positivity, without increasing the overall risk. However, in populations with a low selenium intake, even a slight increase in the UIC to 200~300 µg/L may significantly increase the risk of developing TPOAb positivity. These findings underscore the need for cautious iodine supplementation in selenium-deficient populations. In the high-selenium-intake group, the influence of the UIC on the thyroid antibody levels and their nonlinear relationship both disappeared. We speculate that this was due to the intrinsic interaction between selenium and iodine in the body. This result suggests, from a cross-sectional study perspective, that selenium supplementation might protect the thyroid from autoimmune damage caused by excessive iodine exposure. The comparison of results between the low- and high-selenium groups highlights the necessity of cosupplementation with iodine and selenium. Studies have revealed that the range of selenium intake among U.S. residents is 93 µg (women) to 134 µg (men) [32], which is almost within the range of the moderate selenium intake in this study (88.95 µg to <133.60 µg), where the U-shaped relationship between the UIC and the risk of developing TPOAb positivity was most evident. Similar to the overall study conclusions, the risk of developing TPOAb positivity was lowest when the UIC was 121.47 µg/L in the moderate-selenium-intake subgroup. Therefore, given the current selenium intake status of U.S. residents, the risk of developing TPOAb positivity did not increase when the UIC was <300 µg/L. For TgAb antibody positivity, there was no significant interaction between selenium intake and the UIC. This may be because there is a certain nonlinear relationship between selenium intake and the risk of developing TgAb positivity, and the interaction with the UIC might be obscured. However, we still found a potential protective effect of a high selenium intake on the risk of developing TgAb positivity. Given the lack of a significant interaction between the UIC and other confounding factors (besides selenium intake), we speculate that other non-iodine-dependent confounding factors may have a more independent impact on thyroid antibody positivity. In contrast, selenium and iodine are related in terms of both trace element characteristics and the biological mechanisms affecting the thyroid, making the discussion of their interactions more valuable.

Previous studies have reported the relationship between selenium intake and thyroid antibody levels in the NHANES [33], with conclusions similar to ours; the log-transformed selenium intake was significantly negatively correlated with the TPOAb levels and nonlinearly related to the TgAb levels [33]. However, in our study, the association between selenium and the thyroid antibody levels disappeared after adjusting for confounding factors. This may be due to differences in the study populations included in the two studies. Our study included only adults aged 20 years and older, whereas the study by Zheng et al. also included adolescents aged 12 years and older [33]. We speculate that environmental factors such as age, sex, smoking status, and alcohol consumption status have a far greater impact on thyroid antibody levels in adults than does dietary selenium intake, so the relationship between selenium and thyroid antibody levels became nonsignificant after controlling for confounding factors. Additionally, there are differences in the definition of TgAb positivity between the two studies, which may partially explain the differences. A large cross-sectional survey in China reported that, when the UIC is <100 µg/L, the risk of developing TPOAb positivity significantly increases [3]. Although our logistic regression analysis did not reveal a difference in the risk of developing TPOAb positivity between individuals with a UIC <100 µg/L and those with an adequate UIC, the U-shaped relationship shown by the RCS curve suggests a potential association between a low UIC and an increased risk of developing TPOAb positivity. Compared with that used in the study by Teng et al., our stratification of the UIC was more detailed, with a broader safe range [3].

Based on this study and previous research, a consistent conclusion can be drawn regarding the U-shaped relationship between urinary iodine and thyroid autoantibodies. Therefore, public health policies should manage the upper and lower limits of iodine intake with greater precision. When supplementing iodine, a sufficient selenium intake should also be ensured. The recommended intake of iodine and selenium should be tailored to the local nutritional status, considering the varying levels of iodine and selenium intake across different regions. For instance, in areas with low selenium levels, it is advisable to limit the recommended urinary iodine level to no more than 200 µg/L. Additionally, in low-selenium areas, public health authorities should consider promoting selenium intake through food fortification or supplementation to reduce the risk of thyroid-related diseases. Future research should employ longitudinal study designs to clarify causal relationships, such as conducting regional cohort studies in areas with varying iodine and selenium nutrition profiles (e.g., high-iodine, high-selenium areas; high-iodine, low-selenium areas). Selenium supplementation interventions in high-iodine areas should also be explored to provide important evidence for developing more precise public health policies.

There are several limitations to this study. First, owing to the inclusion criteria and data completeness limitations, our results may not fully represent the entire adult population aged 20 years and older in the U.S. Second, the selenium intake calculated based on individual dietary recall and supplement use is susceptible to recall bias. Third, for AITD, we lacked relevant ultrasound results, so we could not explore the relationship between the UIC and the risk of developing AITD. The cross-sectional nature of the data limits our ability to determine whether the exposure preceded the outcome over time. Therefore, our results can only describe associations and cannot make definitive causal inferences. Although we adjusted for known confounders in the statistical analysis, we cannot entirely rule out the potential impact of unmeasured confounding variables. Finally, to retain the sensitivity of our findings and better detect potential trends and interpret the practical significance of effects (rather than just the statistical significance), we did not apply more conservative multiple testing corrections for the *p*-values. Therefore, the causal relationship among iodine, selenium, and thyroid antibodies requires further prospective studies to obtain more robust conclusions that can be applied to public health and clinical practice.

## 5. Conclusions

In this study, we detected a U-shaped relationship between the UIC and the risk of developing TPOAb positivity. A UIC of 500 µg/L~800 µg/L was an independent risk factor for the development of thyroid antibody positivity, and there was an interaction between selenium intake and the UIC. A high selenium intake can protect the thyroid from autoimmune damage caused by excessive iodine.

## Figures and Tables

**Table 1:** Baseline characteristics of the subjects stratified by the UIC.

		UIC (μg/L)					*p*-Value ^2^
Characteristic	Total *n* = 6612 (100%) ^1^	<100 *n* = 2140 (34%) ^1^	100 to <200 *n* = 2227 (32%) ^1^	200 to <300 *n* = 1131 (17%) ^1^	300 to <500 *n* = 826 (12%) ^1^	500 to ≤800 *n* = 288 (4.0%) ^1^	
Age (years)	46 (33, 59)	45 (32, 56)	46 (33, 59)	48 (35, 61)	48 (34, 61)	50 (35, 62)	0.015
Sex							<0.001
Men	3365 (48.11%)	976 (41.83%)	1127 (48.38%)	631 (55.97%)	469 (53.06%)	162 (51.06%)	
Women	3247 (51.89%)	1164 (58.17%)	1100 (51.62%)	500 (44.03%)	357 (46.94%)	126 (48.94%)	
Race							0.195
Non-Hispanic White	3193 (71.68%)	1020 (70.88%)	1044 (71.43%)	560 (72.72%)	421 (73.92%)	148 (69.26%)	
Non-Hispanic Black	1307 (10.01%)	473 (10.96%)	440 (10.24%)	215 (9.54%)	129 (7.62%)	50 (9.38%)	
Mexican American	1046 (7.86%)	297 (6.97%)	378 (8.27%)	191 (8.68%)	132 (7.67%)	48 (9.34%)	
Other Hispanic	671 (5.08%)	200 (4.99%)	229 (4.76%)	121 (5.74%)	92 (5.14%)	29 (5.44%)	
Other/multiracial	395 (5.37%)	150 (6.19%)	136 (5.31%)	44 (3.32%)	52 (5.65%)	13 (6.58%)	
Education attainment							0.404
Less than 9th grade	727 (5.56%)	186 (4.29%)	242 (5.48%)	156 (7.57%)	105 (6.10%)	38 (6.88%)	
9–11th grade	1090 (12.15%)	350 (11.77%)	362 (11.83%)	198 (13.46%)	129 (12.23%)	51 (12.10%)	
High school grad/GED	1546 (23.92%)	531 (24.72%)	503 (23.28%)	251 (25.18%)	194 (21.56%)	67 (24.24%)	
Some college or AA degree	1837 (30.51%)	608 (30.52%)	647 (31.84%)	292 (27.74%)	211 (30.07%)	79 (32.72%)	
College graduate or above	1412 (27.87%)	465 (28.70%)	473 (27.57%)	234 (26.05%)	187 (30.04%)	53 (24.07%)	
Ratio of family income to poverty							0.009
≤1.3	2023 (21.45%)	653 (20.70%)	660 (20.97%)	334 (21.24%)	268 (22.91%)	108 (28.14%)	
1.3~3.5	2497 (33.97%)	791 (33.35%)	830 (33.58%)	468 (40.21%)	309 (30.91%)	99 (25.46%)	
≥3.5	2092 (44.58%)	696 (45.95%)	737 (45.44%)	329 (38.54%)	249 (46.18%)	81 (46.40%)	
Smoking status							0.002
Current smoker	1431 (22.40%)	526 (25.99%)	466 (22.47%)	231 (19.80%)	156 (17.83%)	52 (16.29%)	
Former smoker	1715 (24.70%)	514 (24.60%)	583 (23.81%)	314 (26.43%)	219 (22.71%)	85 (31.67%)	
Non-smoker	3466 (52.90%)	1100 (49.41%)	1178 (53.72%)	586 (53.77%)	451 (59.47%)	151 (52.03%)	
Alcohol consumption							0.262
1–5 drinks/month	3266 (49.35%)	1014 (46.60%)	1095 (49.36%)	584 (49.96%)	427 (55.27%)	146 (51.77%)	
5–10 drinks/month	531 (9.66%)	192 (10.19%)	175 (10.21%)	83 (8.72%)	62 (8.49%)	19 (8.30%)	
10+ drinks/month	1005 (18.72%)	366 (21.26%)	342 (18.96%)	148 (17.20%)	107 (14.64%)	42 (14.15%)	
Non-drinker	1809 (22.26%)	567 (21.92%)	615 (21.47%)	316 (24.12%)	230 (21.60%)	81 (25.78%)	
BMI (kg/m^2^)							0.001
Underweight (<18.5)	101 (1.53%)	46 (1.71%)	27 (1.32%)	16 (1.67%)	7 (0.92%)	5 (2.89%)	
Normal (18.5 to <25)	1812 (30.20%)	727 (37.21%)	556 (27.23%)	250 (24.32%)	203 (27.28%)	76 (28.34%)	
Overweight (25 to <30)	2270 (33.70%)	695 (31.57%)	807 (35.29%)	390 (35.35%)	277 (33.26%)	101 (33.39%)	
Obese (30 or greater)	2429 (34.57%)	672 (29.51%)	837 (36.16%)	475 (38.65%)	339 (38.54%)	106 (35.38%)	
TSH (mIU/L)	2.11 (3.40)	2.04 (3.02)	2.10 (3.98)	2.93 (1.59)	3.06 (1.71)	2.96 (1.79)	0.211
fT4 (pmol/L)	10.26 (2.00)	10.33 (2.01)	10.33 (1.91)	2.35 (4.06)	1.99 (1.77)	2.04 (1.45)	0.250
fT3 (pg/mL)	3.18 (0.43)	3.19 (0.36)	3.20 (0.52)	10.10 (1.96)	10.10 (2.16)	10.19 (2.12)	0.302
TPOAb-positive	736 (11.98%)	237 (12.19%)	219 (10.59%)	148 (13.58%)	96 (11.74%)	36 (15.56%)	0.201
TgAb-positive	461 (7.49%)	157 (7.51%)	139 (7.59%)	84 (6.55%)	55 (6.23%)	26 (14.32%)	0.029
Selenium (μg)	126.55 (66.48)	119.93 (64.46)	126.43 (61.80)	133.73 (69.56)	135.23 (78.71)	126.99 (59.29)	<0.001

^1^ Median (IQR) or mean (SE) for continuous variables; weighted % for categorical variables. ^2^ Chi-squared test with Rao and Scott’s second-order correction; Wilcoxon rank-sum test for complex survey samples. BMI, body mass index; fT3, free triiodothyronine; fT4, free thyroxine; GED, general educational development test; TgAb, thyroglobulin antibody; TPOAb, thyroid peroxidase antibody; TSH, thyroid-stimulating hormone; UIC, urinary iodine concentration.

**Table 2:** Weighted multinomial logistic regression analysis of the associations of the UIC category with TPOAb positivity, TgAb positivity, and TAI risk.

UIC (μg/L)	Model 1			Model 2			Model 3		
	OR	(95% CI)	*p*-Value	OR	(95% CI)	*p*-Value	OR	(95% CI)	*p*-Value
TPOAb Positive									
UIC < 100	1.17	(0.87, 1.57)	0.280	1.16	(0.86, 1.57)	0.314	1.17	(0.85, 1.60)	0.322
100 ≤ UIC < 200	Ref.	Ref.	-	Ref.	Ref.	-	Ref.	Ref.	-
200 ≤ UIC < 300	1.33	(0.93, 1.90)	0.119	1.37	(0.95, 1.98)	0.086	1.42	(0.96, 2.08)	0.074
300 ≤ UIC < 500	1.12	(0.83, 1.51)	0.441	1.14	(0.84, 1.55)	0.381	1.13	(0.83, 1.54)	0.431
500 ≤ UIC ≤ 800	**1.56**	**(1.05, 2.31)**	**0.029**	**1.55**	**(1.06, 2.28)**	**0.027**	**1.57**	**(1.07, 2.30)**	**0.022**
TgAb Positive									
UIC < 100	0.99	0.71, 1.37	0.945	1.00	0.72, 1.39	0.988	1.00	0.70, 1.42	0.991
100 ≤ UIC < 200	Ref.	Ref.	-	Ref.	Ref.	-	Ref.	Ref.	-
200 ≤ UIC < 300	0.85	0.54, 1.34	0.486	0.86	0.55, 1.34	0.493	0.87	0.54, 1.38	0.531
300 ≤ UIC < 500	0.81	0.55, 1.18	0.264	0.81	0.56, 1.17	0.249	0.78	0.54, 1.14	0.189
500 ≤ UIC ≤ 800	**2.03**	**(1.14, 3.62)**	**0.017**	**2.01**	**(1.13, 3.57)**	**0.019**	**2.00**	**(1.10, 3.65)**	**0.025**
TAI									
UIC < 100	1.11	0.87, 1.42	0.393	1.11	0.86, 1.42	0.421	1.09	0.84, 1.43	0.495
100 ≤ UIC < 200	Ref.	Ref.	-	Ref.	Ref.	-	Ref.	Ref.	-
200 ≤ UIC < 300	1.16	0.83, 1.61	0.370	1.20	0.86, 1.66	0.277	1.22	0.86, 1.73	0.246
300 ≤ UIC < 500	1.09	0.84, 1.42	0.513	1.11	0.85, 1.44	0.430	1.10	0.84, 1.43	0.484
500 ≤ UIC ≤ 800	**1.61**	**(1.07, 2.44)**	**0.024**	**1.61**	**(1.08, 2.42)**	**0.022**	**1.62**	**(1.07, 2.45)**	**0.024**

*p*-values were calculated via a weighted logistic regression analysis. Model 1: This model was not adjusted for any covariates. Model 2: Adjusted for age and sex. Model 3: Adjusted for age, sex, race, BMI, household income, education level, smoking status, alcohol consumption status, and selenium intake. BMI, body mass index; CI, confidence interval; OR, odds ratio; TAI, thyroid autoimmunity; TgAb, thyroglobulin antibody; TPOAb, thyroid peroxidase antibody; UIC, urinary iodine concentration. Bold: *p* < 0.05.

**Table 3:** Baseline characteristics of subjects stratified by selenium intake.

Characteristic	Overall, N = 6612 (100%) ^1^	Selenium (μg)			*p*-Value ^2^
		T1, N = 2204 (31%) ^1^	T2, N = 2205 (33%) ^1^	T3, N = 2203 (37%) ^1^	
Age (year)	46 (33, 59)	46 (33, 60)	47 (34, 59)	45 (32, 57)	0.030
Sex					<0.001
Men	3365 (48.11%)	695 (26.31%)	1097 (45.85%)	1573 (68.37%)	
Women	3247 (51.89%)	1509 (73.69%)	1108 (54.15%)	630 (31.63%)	
Race					0.004
Non-Hispanic White	3193 (71.68%)	997 (70.55%)	1035 (69.29%)	1161 (74.78%)	
Non-Hispanic Black	1307 (10.01%)	491 (11.78%)	437 (10.60%)	379 (7.99%)	
Mexican American	1046 (7.86%)	373 (7.72%)	360 (8.61%)	313 (7.31%)	
Other Hispanic	671 (5.08%)	247 (5.52%)	226 (5.40%)	198 (4.41%)	
Other/multiracial	395 (5.37%)	96 (4.43%)	147 (6.09%)	152 (5.51%)	
Education attainment					<0.001
Less than 9th grade	727 (5.56%)	329 (7.77%)	229 (5.35%)	169 (3.89%)	
9–11th grade	1090 (12.15%)	420 (14.60%)	339 (11.38%)	331 (10.79%)	
High school grad/GED	1546 (23.92%)	547 (25.34%)	505 (24.35%)	494 (22.34%)	
Some college or AA degree	1837 (30.51%)	564 (29.82%)	644 (30.88%)	629 (30.75%)	
College graduate or above	1412 (27.87%)	344 (22.47%)	488 (28.04%)	580 (32.22%)	
Household income					<0.001
≤1.3	2023 (21.45%)	802 (26.57%)	653 (21.07%)	568 (17.51%)	
1.3~3.5	2497 (33.97%)	860 (36.98%)	839 (34.02%)	798 (31.40%)	
≥3.5	2092 (44.58%)	542 (36.44%)	713 (44.91%)	837 (51.09%)	
Smoking status					<0.001
Current smoker	1431 (22.40%)	514 (26.33%)	455 (20.91%)	462 (20.45%)	
Former smoker	1715 (24.70%)	519 (20.69%)	547 (25.23%)	649 (27.58%)	
Non-smoker	3466 (52.90%)	1171 (52.98%)	1203 (53.86%)	1092 (51.97%)	
Alcohol consumption					<0.001
1–5 drinks/month	3266 (49.35%)	1070 (49.88%)	1088 (47.99%)	1108 (50.12%)	
5–10 drinks/month	531 (9.66%)	138 (7.65%)	176 (9.20%)	217 (11.76%)	
10+ drinks/month	1005 (18.72%)	215 (13.10%)	349 (19.89%)	441 (22.37%)	
Non-drinker	1809 (22.26%)	781 (29.37%)	592 (22.93%)	436 (15.72%)	
BMI (kg/m^2^)					0.327
Underweight (<18.5)	101 (1.53%)	45 (2.13%)	23 (0.75%)	33 (1.72%)	
Normal (18.5 to <25)	1812 (30.20%)	564 (30.17%)	613 (30.44%)	635 (30.01%)	
Overweight (25 to <30)	2270 (33.70%)	766 (34.14%)	762 (33.57%)	742 (33.45%)	
Obesity (30 or greater)	2429 (34.57%)	829 (33.56%)	807 (35.23%)	793 (34.82%)	
TSH (mIU/L)	2.11 (3.40)	2.12 (3.45)	2.03 (2.31)	2.16 (4.10)	0.423
fT4 (pmol/L)	10.26 (2.00)	10.33 (2.07)	10.24 (2.02)	10.22 (1.91)	0.502
fT3 (pg/mL)	3.18 (0.43)	3.16 (0.41)	3.17 (0.49)	3.21 (0.38)	0.006
TPOAb-positive	73 (11.98%)	269 (13.02%)	249 (12.51%)	218 (10.64%)	0.129
TgAb-positive	46 (7.49%)	177 (8.95%)	157 (8.36%)	127 (5.48%)	0.011
UIC (μg/L)	179.93 (139.26)	166.28 (135.83)	182.15 (142.31)	189.34 (138.48)	<0.001

^1^ Median (IQR) or mean (SE) for continuous variables; weighted % for categorical variables. ^2^ Chi-squared test with Rao and Scott’s second-order correction; Wilcoxon rank-sum test for complex survey samples. T1: <88.95 µg; T2: 88.95 µg to <133.60 µg; T3: ≥133.60 µg. BMI, body mass index; fT3, free triiodothyronine; fT4, free thyroxine; GED, general educational development test; TgAb, thyroglobulin antibody; TPOAb, thyroid peroxidase antibody; TSH, thyroid-stimulating hormone; UIC, urinary iodine concentration.

**Table 4:** Associations of selenium intake with TPOAb and TgAb levels.

	**Model 1**		**Model 2**		**Model 3**	
	**β (95% CI)**	***p***	**β (95% CI)**	***p***	**β (95% CI)**	***p***
TPOAb	**−0.049 (−0.092, −0.005)**	**0.028**	−0.007 (−0.055, 0.040)	0.765	−0.025 (−0.068, 0.019)	0.257
TgAb	−0.025 (−0.050, 0.000)	0.054	−0.015 (−0.048, 0.017)	0.353	−0.018 (−0.049, 0.014)	0.257
	**Model 1**		**Model 2**		**Model 3**	
	**OR (95% CI)**	***p***	**OR (95% CI)**	***p***	**OR (95% CI)**	***p***
TPOAb positive						
T1	Ref.	-	Ref.	-	Ref.	-
T2	0.995 (0.969, 1.021)	0.694	1.009 (0.983, 1.035)	0.489	0.999 (0.968, 1.032)	0.967
T3	0.976 (0.952, 1.002)	0.068	1.009 (0.980, 1.039)	0.543	0.988 (0.943, 1.035)	0.598
TgAb positive						
T1	Ref.	-	Ref.	-	Ref.	-
T2	0.994 (0.971, 1.018)	0.621	1.000 (0.975, 1.026)	0.975	1.000 (0.972, 1.030)	0.978
T3	**0.966 (0.941, 0.992)**	**0.012**	0.981 (0.952, 1.011)	0.206	0.985 (0.945, 1.027)	0.473

*p*-values were calculated via a weighted linear regression or weighted logistic regression analysis. Model 1: This model was not adjusted for any covariates. Model 2: Adjusted for age and sex. Model 3: Adjusted for age, sex, race, BMI, household income, education level, smoking status, alcohol consumption status, and selenium intake. BMI, body mass index; CI, confidence interval; OR, odds ratio; TgAb, thyroglobulin antibody; TPOAb, thyroid peroxidase antibody. Bold: *p* < 0.05.

**Table 5:** Interaction effect between the UIC and selenium intake on thyroid antibody positivity.

	Model 1			*p* for Interaction	Model 2			*p* for Interaction	Model 3			*p* for Interaction
	OR (95% CI)		*p*		OR (95% CI)		*p*		OR (95% CI)		*p*	
**TPOAb Positive**				0.067 *				0.087 *				0.097 *
**Selenium *T1***												
UIC < 100	1.39 (0.85, 2.29)		0.185		1.43 (0.88, 2.32)		0.148		1.37 (0.85, 2.22)		0.185	
100 ≤ UIC < 200	Ref.		-		Ref.		-		Ref.		-	
200 ≤ UIC < 300	**1.83 (1.01, 3.32)**		**0.046**		**1.88 (1.02, 3.47)**		**0.043**		**1.91 (1.01, 3.61)**		**0.046**	
300 ≤ UIC < 500	**0.60 (0.36, 1.00)**		**0.049**		0.62 (0.36, 1.07)		0.086		0.60 (0.34, 1.04)		0.065	
500 ≤ UIC < 800	1.12 (0.42, 3.02)		0.817		1.13 (0.42, 3.00)		0.808		1.17 (0.44, 3.14)		0.745	
**Selenium *T2***												
UIC < 100	1.14 (0.72, 1.80)		0.558		1.10 (0.70, 1.74)		0.665		1.09 (0.68, 1.74)		0.706	
100 ≤ UIC < 200	Ref.		-		Ref.		-		Ref.		-	
200 ≤ UIC < 300	1.09 (0.70, 1.70)		0.693		1.13 (0.74, 1.72)		0.566		1.16 (0.75, 1.79)		0.479	
300 ≤ UIC < 500	**1.90 (1.17, 3.06)**		**0.010**		**1.93** (**1.18, 3.15**)		**0.010**		**1.82** (**1.11, 2.97**)		**0.019**	
500 ≤ UIC < 800	**2.45 (1.29, 4.64)**		**0.007**		**2.45** (**1.30, 4.60**)		**0.006**		**2.60** (**1.46, 4.63**)		**0.002**	
**Selenium *T3***												
UIC < 100	0.96 (0.61, 1.51)		0.848		1.03 (0.64, 1.66)		0.910		1.12 (0.70, 1.79)		0.635	
100 ≤ UIC < 200	Ref.		-		Ref.		-		Ref.		-	
200 ≤ UIC < 300	1.20 (0.70, 2.06		0.507		1.25 (0.72, 2.19)		0.418		1.33 (0.74, 2.41)		0.329	
300 ≤ UIC < 500	0.96 (0.62, 1.48)		0.842		0.96 (0.59, 1.55)		0.855		0.96 (0.59, 1.58)		0.877	
500 ≤ UIC < 800	1.12 (0.48, 2.60)		0.782		1.11 (0.46, 2.67)		0.817		1.13 (0.47, 2.68)		0.780	
**TgAb Positive**				0.335				0.352				0.340
**TAI**				0.235				0.264				0.283

*p*-values were calculated via a weighted logistic regression analysis. Model 1: This model was not adjusted for any covariates. Model 2: Adjusted for age and sex. Model 3: Adjusted for age, sex, race, BMI, household income, education level, smoking status, alcohol consumption status, and selenium intake. BMI, body mass index; CI, confidence interval; OR, odds ratio; TAI, thyroid autoimmunity; TgAb, thyroglobulin antibody; TPOAb, thyroid peroxidase antibody; UIC, urinary iodine concentration. Bold: *p* < 0.05. *: *p* for interaction < 0.10.

## Data Availability

The data are available on the NHANES website (http://www.cdc.gov/nchs/nhanes.htm, accessed on 10 March 2024).

## Supplementary Materials

The following supporting information can be downloaded at: https://www.mdpi.com/article/10.3390/nu16203443/s1, Table S1: Characteristics of NHANES participants. Table S2: Interaction effect between the UIC and other factors on thyroid antibody positivity.
