# Supplementary material for: The Relationships among the Urinary Iodine Concentration, Selenium Intake, and Thyroid Antibodies in Adults, Including the Interaction between Iodine and Selenium: National Health and Nutrition Examination Survey 2007–2012
Source: Nutrients. 2024 Oct 11;16(20):3443. doi: 10.3390/nu16203443 (PMC11509967; doi:10.3390/nu16203443)
Supplement: Supplementary file 1 [file nutrients-16-03443-s001.zip › nutrients-3246521-supplementary.pdf]

Supplementary Table S1. Characteristics of NHANES participants.

**Characteristic**	**N**[1]	**All participants aged≥20 years** n = 15,983 (70%)[2]	**Participants aged≥20 years and excluded in this study** n = 9,371 (58%)[2]	**Participants aged≥20 years and included in this study** n = 6,612 (30%)[2]	***p*-Value**[3]	***p*-Value**[4]
**Age (year)**	15,983	46 (33, 59)	46 (33, 60)	46 (33, 59)	0.339	0.337
**Sex**	15,983				0.856	0.856
Men		7,845 (48.01%)	4,480 (47.95%)	3,365 (48.11%)		
Women		8,138 (51.99%)	4,891 (52.05%)	3,247 (51.89%)		
**Race**	15,983				**0.021**	**0.021**
Non-Hispanic White		7,176 (68.44%)	3,983 (66.08%)	3,193 (71.68%)		
Non-Hispanic Black		3,435 (11.39%)	2,128 (12.40%)	1,307 (10.01%)		
Mexican American		2,459 (8.28%)	1,413 (8.58%)	1,046 (7.86%)		
Other Hispanic		1,645 (5.45%)	974 (5.72%)	671 (5.08%)		
Other/multiracial		1,268 (6.45%)	873 (7.23%)	395 (5.37%)		
**Education attainment**	15,963				0.330	0.328
9-11th Grade		2,551 (12.42%)	1,461 (12.61%)	1,090 (12.15%)		

College Graduate or above		3,412 (27.80%)	2,000 (27.75%)	1,412 (27.87%)		
High School Grad/GED		3,665 (22.69%)	2,119 (21.80%)	1,546 (23.92%)		
Less Than 9th Grade		1,850 (5.96%)	1,123 (6.26%)	727 (5.56%)		
Some College or AA degree		4,485 (31.13%)	2,648 (31.59%)	1,837 (30.51%)		
**Ratio of family income to poverty**	14,597				**0.046**	**0.047**
<=1.3		4,846 (23.16%)	2,823 (24.57%)	2,023 (21.45%)		
1.3~3.5		5,386 (34.37%)	2,889 (34.69%)	2,497 (33.97%)		
>=3.5		4,365 (42.48%)	2,273 (40.74%)	2,092 (44.58%)		
**Smoke status**	15,977				0.167	0.174
Current smoker		3,426 (21.52%)	1,995 (20.88%)	1,431 (22.40%)		
Former smoker		3,886 (24.32%)	2,171 (24.05%)	1,715 (24.70%)		
Never smoker		8,665 (54.16%)	5,199 (55.08%)	3,466 (52.90%)		
**Alcohol consumption**	14,843				0.995	0.995
1-5 drinks/month		7,345 (49.32%)	4,079 (49.30%)	3,266 (49.35%)		
5-10 drinks/month		1,179 (9.70%)	648 (9.72%)	531 (9.66%)		

10+ drinks/month		2,224 (18.76%)	1,219 (18.80%)	1,005 (18.72%)		
Non-drinker		4,093 (22.21%)	2,284 (22.18%)	1,809 (22.26%)		
wait		2 (0.01%)	1 (0.01%)	1 (0.01%)		
**BMI (kg/m^2)**	15,799				0.672	0.665
Underweight (<18.5)		260 (1.66%)	159 (1.77%)	101 (1.53%)		
Normal (18.5 to <25)		4,358 (29.58%)	2,546 (29.12%)	1,812 (30.20%)		
Overweight (25 to <30)		5,293 (33.56%)	3,023 (33.45%)	2,270 (33.70%)		
Obese (30 or greater)		5,888 (35.20%)	3,459 (35.66%)	2,429 (34.57%)		
**TSH (mIU/L)**	8,344	2.13 (3.42)	2.26 (3.53)	2.11 (3.40)	0.501	0.500
**fT4 (pmol/L)**	8,344	10.25 (2.06)	10.20 (2.31)	10.26 (2.00)	**0.040**	**0.034**
**fT3 (pg/mL)**	8,337	3.17 (0.44)	3.12 (0.46)	3.18 (0.43)	**<0.001**	**<0.001**
**TPOAb positive**	8,265	924.00 (12.02%)	188.00 (12.18%)	736.00 (11.98%)	0.885	0.884
**TgAb positive**	8,293	622.00 (8.00%)	161.00 (10.33%)	461.00 (7.49%)	0.117	0.083
**Selenium (µg)**	15,983	130.76 (550.45)	133.82 (721.59)	126.55 (66.48)	0.546	0.547
**UIC (µg/L)**	8,578	309.43 (6,688.11)	820.86 (14,868.47)	179.93 (139.26)	**<0.001**	**<0.001**

[1]n not Missing (unweighted)

[2]median (IQR) for continuous; n (%) for categorical
[3]chi-squared test with Rao & Scott's second-order correction; Wilcoxon rank-sum test for complex survey samples; p-value: compared with all participants aged≥20 years
[4]chi-squared test with Rao & Scott's second-order correction; Wilcoxon rank-sum test for complex survey samples; p-value: compared with Participants aged≥20 years and excluded in this study≥20 years

BMI, body mass index; fT3, free triiodothyronine; fT4, free thyroxine; GED, general educational development test; TgAb, thyroglobulin antibody; TPOAb, thyroid peroxidase antibody; TSH, thyroid-stimulating hormone; UIC, urinary iodine concentration.

Supplementary Table S2. Interaction effect between the UIC and other factors on thyroid antibody positivity.

**TPOAb Positive**		**Model1**	**Model2**	**Model3**
		**p for Interaction**	**p for Interaction**	**p for Interaction**
	Age	0.925	0.923	0.946
	Sex	0.893	0.865	0.878
	Race	0.506	0.569	0.618
	BMI	0.14	0.179	0.221
	Education	0.4	0.43	0.477
	Smoking status	0.813	0.854	0.874
	Alcohol consumption	0.594	0.601	0.588
	Ratio of family income to poverty	0.869	0.757	0.728
	Selenium	**0.067**	**0.087**	**0.097**
**TgAb Positive**		**Model1**	**Model2**	**Model3**
		**p for Interaction**	**p for Interaction**	**p for Interaction**
	Age	0.678	0.698	0.733
	Sex	0.572	0.528	0.496
	Race	0.282	0.32	0.366
	BMI	0.288	0.35	0.44
	Education	0.418	0.396	0.426
	Smoking status	0.334	0.379	0.399
	Alcohol consumption	0.146	0.132	0.169
	Ratio of family income to poverty	0.570	0.54	0.591

	Selenium	0.335	0.352	0.34
**TAI**		**Model1**	**Model2**	**Model3**
		**p for Interaction**	**p for Interaction**	**p for Interaction**
	Age	0.795	0.822	0.866
	Sex	0.953	0.921	0.907
	Race	0.4	0.43	0.466
	BMI	0.203	0.265	0.321
	Education	0.425	0.429	0.482
	Smoking status	0.837	0.908	0.921
	Alcohol consumption	0.357	0.357	0.395
	Ratio of family income to poverty	0.923	0.877	0.867
	Selenium	0.235	0.264	0.283

Model 1: This model was not adjusted for any covariates. Model 2: Adjusted for age and sex. Model 3: Adjusted for age, sex, race, BMI, household income, education level, smoking status, alcohol consumption status, and selenium intake. BMI, body mass index; TAI, thyroid autoimmunity; TgAb, thyroglobulin antibody; TPOAb, thyroid peroxidase antibody; Bold: p for interaction < 0.10.
